# Prevalence of pain and use of prescription opioids among older adults: results from the Brazilian Longitudinal Study of Aging (ELSI-Brazil)

**DOI:** 10.1016/j.lana.2023.100459

**Published:** 2023-03-01

**Authors:** Pricila H. Mullachery, Maria Fernanda Lima-Costa, Antônio Ignácio de Loyola Filho

**Affiliations:** a Department of Health Services Administration and Policy, Temple University College of Public Health, Philadelphia, PA, USA; b Instituto René Rachou, Fundação Oswaldo Cruz, Belo Horizonte, MG, Brazil; c Universidade Federal de Minas Gerais, Belo Horizonte, MG, Brazil; d Escola de Enfermagem, Universidade Federal de Minas Gerais, Belo Horizonte, MG, Brazil

**Keywords:** Prescription opioid, Chronic pain, Aging, Brazil

## Abstract

**Background:**

Pain has a significant impact on people's quality of life. The use of prescription opioids to treat pain is associated with an increased risk of opioid use disorders and overdose death. We measured the prevalence of recurrent pain, prescription opioid use, and associations between chronic conditions and prescription opioid use among Brazilian older adults.

**Methods:**

We used data from the first population-based longitudinal study of aging in Brazil (ELSI-Brazil), 2019–2020 (mean age = 63.3; 54.4% female). Outcomes were: (1) experience of recurrent pain and (2) use of opioid analgesics in the past three months among those who experience pain. Exposures included selected health conditions, history of falls, and hospitalizations.

**Findings:**

Prevalence of pain (n = 9234) was 36.9% (95% CI: 32.6–41.1). Pain was reported more frequently by female participants, low-income individuals, and those with a previous diagnosis of arthritis, chronic back pain, depressive symptoms, history of falls, and hospitalizations. Prevalence of opioid use among those reporting pain (n = 3350) was 30% (95% CI: 23.1–38.0). Prevalence of opioid use was higher among female and single individuals. In adjusted models, arthritis, chronic back pain, and presence of depressive symptoms were associated with prescription opioid use.

**Interpretation:**

Prescription opioid use was reported by a sizable portion of the older adults who suffer from pain in Brazil. In a context of growing consumption of prescription opioids, opioid misuse has the potential to increase in the future. Surveillance of prescription opioid use is critical to prevent their harmful consequences.

**Funding:**

ELSI-Brazil was funded by the 10.13039/501100006506Brazilian Ministry of Health.

**Research in context**

**Evidence before this study**

We searched PubMed and SciELO in August 2022 to identify studies examining patterns of opioid analgesic use in Brazil published in English or Portuguese. The studies identified used data from surveillance systems such as the International Narcotics Control Board (INCB) and the National System for Management of Controlled Substances administered by the Brazilian National Health Surveillance Agency (ANVISA), and data from surveys on medication use and substance use. Surveillance data from INCB indicate that opioid consumption in Brazil increased 3-fold between 2000 and 2018. Data from ANVISA showed that codeine represents 98% of the prescriptions, but oxycodone had the largest relative increase between 2009 and 2015. Data on self-reported use of medications from 2014 showed a 0.5% prevalence of prescription opioids among individuals 20–59 years old and 0.8% among those 60 and older. Prevalence of non-medical opioid use was 1.4% in 2015; this prevalence was higher compared to a previous survey (2005), but the majority of individuals reported infrequent use or use for a short time. The opioid crisis in the US and Canada, characterized by widespread prescription of opioid analgesics for chronic pain, offer important lessons to other countries in the region, including Brazil. One of these lessons is that monitoring opioid analgesic use is critical to identifying harmful use of these drugs.

**Added value of this study**

In this study, we characterized the prevalence of recurrent pain and self-reported use of prescription opioids in a nationally representative sample of community-dwelling older adults. Older adults constitute a critical population given their high burden of chronic conditions and medical interventions, which makes them more likely to experience pain compared to the overall population. We found a high prevalence of prescription opioid use; 30% of the older adults who reported recurrent pain said they had used prescription opioids in the past three months. Prescription opioid use was higher among those with arthritis, chronic back pain, and depressive symptoms. Our findings add value to the existing literature and raise awareness of the need for monitoring prescription opioid use for treatment of pain in the region.

**Implications of all the available evidence**

The available evidence points to an increase in prescription opioid use in Brazil in the past two decades, including for medical and non-medical purposes. In the population of older adults who experience recurrent pain, 30% reported opioid use in the past three months. Taken together, the evidence suggests that opioid harm has the potential to increase in the future. There is a need to better understand the frequency and duration of opioid use, particularly among individuals with chronic pain who may be at risk for developing opioid use disorders.

## Introduction

Pain has a significant impact on people's quality of life.^1^ Pain can be caused by chronic conditions such as cancer^2^ and athritis,^3^ or it can be the result of acute processes, trauma, or surgery.^4^^,^^5^ Due to the higher burden of chronic conditions and medical interventions in the elderly population, they are also more likely to experience pain compared to the overall population.^6^

In its various manifestations, experience of pain affects the overall perception of health and is associated with depressive symptoms and low quality of life.^7^ Pain is also associated with lower productivity and exclusion from the workforce.^6^ The impact of pain can be even more severe among individuals with low socioeconomic status^8^ and those working in sectors such as agriculture and service.^9^^,^^10^ In Brazil, population aging and the growing prevalence of chronic diseases^11^ point to a future where a large share of the population will be affected by pain.

Opioid analgesics are part of the World Health Organization's list of essential medicines and have an important role in palliative care and pain relief.^12^ However, the use of these medications is highly concentrated in high-income countries.^13^ There is an unmet need for these medicines in low- and middle-income countries.^14^ Several factors contribute to this gap, including financial and regulatory constraints, concerns about opioid misuse, risk of addiction, and insufficient training of health care providers.^15^

Concerns about opioid misuse are not unfounded. In countries such as the United States and Canada, where the use of prescription opioids is widespread, there is also a high prevalence of opioid use for non-medical purposes, and high levels of opioid use disorders, which in turn are associated with high opioid overdose rates.^16^ For example, the US reported over 68,000 opioid overdose deaths in 2020.^17^ Opioid overdose deaths increased exponentially in the US in the last 30 years,^18^ after the approval of a new formulation of oxycodone, incorrectly defined by the US Food and Drug Administration as a having low risk for addiction.^19^ This new formulation was then broadly advertised by pharmaceutical companies as a safe and effective way for prolonged treatment of severe and moderate pain.^20^^,^^21^

While the unmet need for medications to treat pain in low- and middle-income countries constitutes an important barrier to access to health care, the use of prescription opioids in the treatment of pain should be carefully monitored. In Brazil, surveillance data from the International Narcotics Control Board indicate that prescription opioid consumption increased 3-fold between 2000 and 2018, from 172 daily doses per million people in 2000–2002 to 512 daily doses per million people in 2016–2018.^22^ Opioid analgesics are controlled substances and can only be obtained with a prescription from a physician or a dentist.^23^ Prescribers use a duplicated special prescription form. In addition, pharmacies must register dispensing of medications via the System for Management of Controlled Substances run by the Brazilian National Health Surveillance Agency (ANVISA). Data from this system show a considerable increase in opioid dispensing, with codeine representing 98% of the prescriptions, and oxycodone representing the largest relative increase between 2009 and 2015.^24^ Despite this increase, the use of prescription opioids by the end of that period was relatively low; a national survey of medication use indicated that in 2014 the prevalence of prescription opioid use was 0.5% among individuals 20–59 years old, and 0.8% among those 60 and older. Opioids represented only 1.7% of all pain medication used (vs. 71% and 26% for non-opioid agents and NSAIDs, respectively).^25^ This same survey found that about 70% of the individuals that reported opioid use said they used it for acute or occasional health conditions, and about 30% used it for chronic conditions, with pain being the main reason for use in 62% of the cases.^25^ Non-medical use of prescription opioids was 1.4% in 2015, according to self-reported data.^26^ Taken together, these data points to an increase in prescription opioid use in Brazil, likely driven by changes in prescriber behavior, but also that consumption is still relatively low according to population surveys.

Continued monitoring of prescription opioid use is critical to identify potential overuse of these drugs in Brazil with a focus on different groups of the population that might be at increased risk, including those who suffer from pain associated with chronic diseases, trauma, or surgery. In particular, older adults constitute a critical population given their high burden of chronic conditions and medical interventions, which makes them more likely to experience pain compared to the overall population. In this study, we characterized the prevalence of recurrent pain and the self-reported use of prescription opioids using data from the first nationally representative study of community-dwelling older adults in Brazil. We also measured the association between various chronic conditions and prescription opioid use.

## Methods

### Study design and target population

We used data from the second wave of the Brazilian Longitudinal Study of Aging (or *Estudo Longitudinal de Saúde dos Idosos* *Brasileiros* - ELSI-Brazil), collected via face-to-face interviews between 2019 and 2020. ELSI-Brazil is the first population-based longitudinal study of aging in Brazil and includes a nationally representative sample of community-dwelling older adults (aged 50 or older); its purpose is to examine the dynamics of aging and its determinants. ELSI-Brazil uses a complex sample design with three stages, municipality, census tract, and household. To ensure that the sample represents municipalities of various sizes, four strata of municipalities based on population size were constructed using the Lavellée and Hidirogloui method.**^27^** Selection of geographic units and households was done using the operational database of the Brazilian Institute of Geography and Statistics, the organization that conducts the Brazilian census.

In each selected household, all residents aged 50 years and older were included in the sample. The final sample consisted of 10,000 planned interviews in 70 municipalities across the country. This sample size allows for the detection of a prevalence of 1% with sample error of 0.25% at a level of significance of 95% and an effect design of 1.5. To maintain the representativeness of the sample throughout the waves, the study also included replacement of participants at each new wave. ELSI's second wave had 9949 participants out of the 10,000 approached (response rate = 99%) and was the first wave to include questions about prescription opioid use. Sample weights were created to account for the different probability of selection and different non-response rates. Detailed description of the study design was published elsewhere.^28^^,^^29^

### Outcomes

In this paper, we examined two outcomes, (1) experience of recurrent pain, measured by the question “Do you suffer from pain that occurs frequently?” (Yes/No), and (2) use of prescription opioids among those who responded “yes” to outcome 1, measured by the question “In the last 3 months, have you taken pain medications that require a prescription such as pain relievers that contain codeine or morphine (Fiorional, Tylex, Duramorf, Demerol, Durogesic, OxyContin, Codex, Percodan, Dimorf, Tramadol)?” (Yes/No). Due to a skip pattern in the questionnaire, we were not able to measure prescription opioid use in the full sample of participants, but only among those who reported pain based on question 1.

### Exposures

Exposure variables included selected chronic conditions associated with experience of pain: arthritis, chronic back pain, cancer, and presence of depressive symptoms. Ascertainment of these conditions was based on participant's reporting of a diagnosis by a doctor measured by the question “Have you ever been told by a doctor that you have (insert condition)?”. We also included self-reported events associated with pain and opioid use, i.e., history of falls and total number of hospitalizations for any reason in the 12 months prior to the survey. In addition, we used an indicator of pain severity as an additional exposure. Pain severity was assessed by the question “What is the intensity of the pain most of the time?” with possible responses being mild, moderate, and intense/severe.

### Covariates

Covariates included sociodemographic characteristics represented by binary sex (male and female), age, marital status (married/cohabiting, single, divorced/separated, and widowed), years of formal education (categorized into 0–4, 5–8, 9–11, and 12 or more years), per capita household income (categorized into quintiles), region of residence (one of the five Brazilian regions, i.e., Southeast, South, Center-West, Northeast, and North), and urban (vs. rural) residence.

### Analytical approach

Our analysis was restricted to participants who had complete data for all variables included in this study (n = 9234 or 92.8% of all participants the second wave of ELSI-Brazil) and accounted for sampling design and sample weights. Please refer to the Supplementary Material for a comparison of ELSI-Brazil participants included in the analysis with those excluded due to lack of responses in at least one variable. For all demographic and socioeconomic groups, inclusion in the analytical sample was near to or above 90%.

For the analysis, we first calculated frequencies for sociodemographic and health-related characteristics of the study participants. In this step, we used four 10-year age categories (50–59, 60–69, 70–79, and 80 or older) and the remaining covariates and exposures as described previously. Second, we calculated prevalence rates for the two outcomes: experience of recurrent pain and prescription opioid use. We also calculated prevalence rates by each covariate and exposure. We used Pearson Chi-square statistics with correction for survey design for two-way tables and calculated 95% confidence intervals for the prevalences. For the outcome experience of pain, the analysis included 9234 respondents. For the outcome use of prescription opioids, the analysis included only those who reported pain, i.e., 3350 participants. Third, we used adjusted Poisson regression models to measure the association between exposures and prescription opioid use. For each exposure (chronic condition or event) we constructed a model with the exposure and covariates (*model 1*). We also tested additional models including one of the other five exposures separately. For example, for the exposure “depressive symptoms”, model 1 includes depressive symptoms and covariates; *model 2* includes depressive symptoms, arthritis, and covariates; *model 3* includes depressive symptoms, back pain, and covariates, and so forth, totaling six models for each exposure. The goal of this approach was to assess changes in the magnitude of the association resulting from the addition of other pain-related exposures. We constructed figures with the marginal predicted probabilities of prescription opioid use by sex based on *model 1*. Finally, for prescription opioid use, we also examined the association between pain severity and opioid use and plotted the results in a figure representing the adjusted prevalence ratios of opioid use for individuals with severe and moderate pain (relative to those with mild pain).

We used STATA 17 for all analytical procedures and the *svy* command to account for complex sampling design.

### Ethics approval and informed consent

ELSI-Brazil was approved by the ethics review committee of the Instituto René Rachou, Fundação Oswaldo Cruz, protocol number: CAAE—34649814.3.0000.5091. All survey participants provided consent and signed an informed consent document.

### Role of funding source

The funders had no role in study design, data collection, data analysis, interpretation or preparation of the manuscript.

## Results

The average age of the study population was 63.3 (SD 10.05). Among the 9234 participants, 5480 were female and 3754 were male, representing 54.4% and 45.6% of the participants respectively. The majority of the participants had between 0 and 4 years of formal education (5336 [53.1%] of 9234 participants), 4949 [61.2%] were married or cohabiting, and 7660 [83.5%] lived in an urban area. Most participants lived in two regions, Southeast (41.5%) and Northeast (29.0%), matching the overall population distribution. Regarding chronic conditions, 1854 [18.8%] of the 9234 participants reported arthritis, 3013 [33.5%] reported chronic back pain, 1118 [13.2%] reported depressive symptoms, and 380 [4.4%] reported cancer. Regarding events associated with pain, 1746 [19%] of the 9234 participants reported a fall, and 570 [5.8%] reported one or more hospitalizations in the last 12 months (Table 1).

**Table 1:** Prevalence of pain and use of prescription opioids according to sociodemographic characteristics and health-related exposures, ELSI-Brazil, 2019–2020.

	Prevalence of pain (n = 9234)				Prevalence of prescription opioid use among those who reported pain (n = 3350)			
	N (%)^a^	Prevalence	95% CI	p-value^b^	N (%)^a^	Prevalence	95% CI	p-value^b^
**Sex**								
Female	5480 (54.4)	42.1	[37.4–46.9]	<0.0001	2221 (62.1)	33.6	[25.9–42.3]	0.0003
Male	3754 (45.6)	30.7	[26.4–35.3]		1129 (37.9)	24.1	[17.9–31.7]	
**Age group**								
50–59	2774 (47.1)	36.5	[31.7–41.7]	0.93	988 (46.7)	30.6	[22.1–40.6]	0.58
60–69	3248 (29.4)	37.4	[32.5–42.7]		1185 (29.8)	27.5	[20.6–35.8]	
70–79	2141 (16.0)	36.6	[32.0–41.4]		771 (15.9)	32.0	[23.8–41.4]	
80+	1070 (7.5)	37.5	[31.6–43.7]		406 (7.6)	32.2	[23.8–41.9]	
**Education**								
0–4 years	5336 (53.1)	39.9	[34.8–45.1]	0.0013	2090 (57.4)	29.6	[21.9–38.7]	0.96
5–8 years	1736 (20.3)	35.7	[30.4–41.2]		598 (19.6)	31.0	[23.2–40.1]	
9–11 years	1559 (19.7)	32.8	[27.8–38.3]		495 (17.5)	29.9	[22.6–38.4]	
12 or more	603 (6.9)	29.0	[24.0–34.7]		167 (5.5)	31.0	[21.0–43.2]	
**Marital status**								
Single	1113 (12.5)	36.3	[29.5–43.8]	0.83	386 (12.3)	41.9	[27.8–57.4]	0.014
Married/cohabiting	4949 (61.2)	37.0	[32.7–41.5]		1801 (61.4)	28.1	[21.9–35.4]	
Divorced	1148 (10.8)	34.9	[28.4–42.0]		389 (10.3)	23.2	[15.7–33.0]	
Widowed	2024 (15.5)	38.3	[31.8–45.1]		774 (16.0)	32.4	[24.0–42.1]	
**Household income**								
Q1 (lowest quintile)	1665 (19.9)	45.9	[40.5–51.4]	<0.0001	749 (24.7)	30.4	[22.6–39.5]	0.76
Q2	1766 (20.0)	36.6	[30.9–42.7]		660 (19.8)	30.3	[21.0–41.7]	
Q3	2135 (20.0)	38.7	[33.1–44.7]		804 (21.1)	27.7	[18.7–39.0]	
Q4	1762 (20.1)	35.2	[29.5–41.3]		624 (19.2)	29.1	[19.4–41.2]	
Q5 (highest quintile)	1906 (20.0)	28.0	[23.5–33.0]		513 (15.2)	33.3	[24.6–43.4]	
**Residence**								
Urban	7660 (83.5)	37.4	[33.0–42.0]	0.38	2829 (84.8)	29.9	[23.1–37.7]	0.88
Rural	1574 (16.5)	34.1	[27.1–41.9]		521 (15.2)	30.8	[18.3–47.0]	
**Region**								
North	720 (6.9)	36.5	[26.1–48.3]	0.14	252 (6.8)	21.6	[7.5–48.3]	0.83
Northeast	2562 (29.0)	41.4	[34.3–48.9]		1094 (32.5)	30.4	[17.6–47.2]	
Southeast	3651 (41.5)	33.5	[27.1–40.5]		1123 (37.7)	30.3	[18.8–44.8]	
South	1265 (13.5)	30.2	[20.1–42.7]		384 (11.1)	37.9	[23.1–55.4]	
Center-West	1036 (9.1)	48.1	[34.3–62.2]		497 (11.9)	25.5	[14.9–40.2]	
**Arthritis**								
Yes	1854 (18.8)	64.7	[58.8–70.1]	<0.0001	1156 (33.0)	36.5	[26.9–47.4]	0.002
No	7380 (81.2)	30.4	[26.6–34.6]		2194 (67.0)	26.8	[20.4–34.3]	
**Chronic back pain**								
Yes	3013 (33.5)	63.9	[59.2–68.2]	<0.0001	1895 (58.0)	33.0	[24.3–43.1]	0.095
No	6221 (66.5)	23.3	[19.6–27.4]		1455 (42.0)	25.9	[19.3–33.7]	
**Depressive symptoms**								
Yes	1188 (13.2)	53.7	[46.6–60.6]	<0.0001	632 (19.2)	41.8	[32.7–51.5]	<0.0001
No	8046 (86.8)	34.3	[30.2–38.6]		2718 (80.8)	27.2	[20.3–35.4]	
**Cancer**								
Yes	380 (4.4)	40.3	[27.5–54.6]	0.60	148 (4.8)	30.1	[16.1–49.0]	0.99
No	8854 (95.6)	36.7	[32.4–41.3]		3202 (95.2)	30.0	[22.8–38.3]	
**Fall (last 12 months)**								
Yes	1746 (19.0)	55.9	[50.3–61.4]	<0.0001	947 (28.9)	31.9	[21.3–44.8]	0.61
No	7488 (81.0)	32.4	[28.4–36.6]		2403 (71.1)	29.2	[22.5–37.0]	
**Hospitalizations**								
None	8664 (94.2)	35.9	[31.6–40.4]		3049 (91.7)	29.4	[22.2–37.8]	0.26
One	415 (4.2)	55.1	[43.2–59.0]	<0.0001	214 (5.8)	37.6	[28.3–47.7]	
Two or more	155 (1.6)	55.9	[44.9–66.4]		87 (2.5)	35.8	[20.6–54.5]	
**Intensity of pain**^c^								
Mild	–	–	–		428 (13.7)	12.1	[7.0–20.1]	<0.0001
Moderate	–	–	–		1754 (50.5)	26.3	[18.1–36.5]	
Severe	–	–	–		1168 (35.8)	42.1	[34.2–50.5]	
**Total**	**9234**	**36.9**	**[32.6–41.4]**		**3350**	**30.0**	**[23.1–38.0]**	

a N represents the unadjusted number of participants in each category and % represents the proportion after adjusting for survey weights.

b From Chi-square Pearson tests comparing subgroups for each variable.

c Among those who reported pain (n = 3350).

The prevalence of recurrent pain in this population was 36.9% (95% CI: 32.6–41.4). Among those who reported pain, 1754 [50.5%] of 3350 had moderate pain and 1168 [35.8%] had severe pain. Pain was more prevalent in female participants (vs. male), those with fewer years of education, and those at the bottom quintiles of income, compared to their counterparts. People who reported a diagnosis of arthritis, chronic back pain, and depressive symptoms, as well as those who reported previous falls and hospitalizations, had higher prevalence of pain compared to those who did not report those conditions or events. Experience of pain did not differ across age groups (Table 1).

The prevalence of prescription opioids among those who reported pain was 30% (95% CI: 23.1–38.0). Prevalence of prescription opioid use was higher among female (vs. male) and single individuals (vs. other marital status). The prevalence was also higher among those with arthritis and depressive symptoms, and those who reported severe and moderate pain (vs. those with mild pain) (Table 1).

Adjusted models showed that arthritis (PR = 1.36; 95% CI: 1.10–1.67), chronic back pain (PR = 1.30; 95% CI: 1.02–1.66), and presence of depressive symptoms (PR = 1.48; 95% CI: 1.23–1.78) were associated with prescription opioid use. For arthritis and depressive symptoms, associations were robust regardless of the inclusion of other chronic conditions or events in models 2–7. Cancer, history of falls and hospitalizations were not associated with higher prescription opioid use (Table 2).

**Table 2:** Associations between chronic conditions or events and prescription opioid use, ELSI-Brazil, 2019–2020.

	Model 1 (main exposure + covariates)	Model 2 (model 1 + arthritis)	Model 3 (model 1 + back pain)	Model 4 (model 1 + depressive symptoms)	Model 5 (model 1 + cancer)	Model 6 (model 1 + fall)	Model 7 (model 1 + hospitalization)
Arthritis	1.36	–	1.30	1.32	1.36	1.36	1.36
95% CI	1.10–1.67		1.08–1.56	1.08–1.62	1.11–1.67	1.10–1.67	1.10–1.68
p-value	**0.004**		**0.006**	**0.008**	**0.004**	**0.004**	**0.004**
Chronic back pain	1.30	1.23	–	1.26	1.30	1.30	1.30
95% CI	1.02–1.66	0.98–1.55		0.99–1.60	1.02–1.66	1.02–1.66	1.02–1.66
p-value	**0.034**	0.076		0.057	**0.033**	**0.033**	**0.033**
Depressive symptoms	1.48	1.44	1.44	–	1.48	1.47	1.46
95% CI	1.23–1.78	1.19–1.73	1.20–1.72		1.23–1.78	1.23–1.76	1.21–1.77
p-value	**<0.0001**	**0.0002**	**0.0001**		**<0.0001**	**<0.0001**	**0.0001**
Cancer	0.99	1.01	1.02	0.94	–	0.99	0.97
95% CI	0.53–1.86	0.56–1.82	0.56–1.85	0.52–1.68		0.52–1.86	0.53–1.78
p-value	0.985	0.980	0.948	0.827		0.971	0.917
Fall	1.07	1.06	1.07	1.05	1.07	–	1.06
95% CI	0.80–1.44	0.78–1.43	0.80–1.43	0.77–1.42	0.80–1.44		0.78–1.43
p-value	0.641	0.707	0.657	0.764	0.639		0.707
Hospitalizations							
One vs. none	1.28	1.30	1.26	1.24	1.29	1.28	–
95% CI	0.94–1.76	0.96–1.76	0.92–1.74	0.90–1.69	0.94–1.75	0.93–1.75	
p-value	0.122	0.094	0.147	0.186	0.110	0.134	
Two or more vs. none	1.24	1.23	1.29	1.12	1.24	1.22	
95% CI	0.77–1.97	0.74–2.05	0.82–2.02	0.71–1.78	0.77–1.98	0.74–2.00	
p-value	0.374	0.418	0.271	0.624	0.372	0.430	

For each chronic condition or event, model 1 includes only covariates, Model 2 includes covariates and arthritis, Model 3 includes covariates and chronic back pain, Model 4 includes covariates and depressive symptoms, Model 5 includes covariates and cancer, Model 6 includes covariates and fall in the past 12 months, Model 7 includes covariates and hospitalizations in the past 12 months, Coefficients were transformed to represent prevalence ratios. 95% CI = 95% Confidence Interval, Covariates were binary sex, age, education, household income categorized into quintiles, urban/rural, and region of residence.

**Fig. 1:**
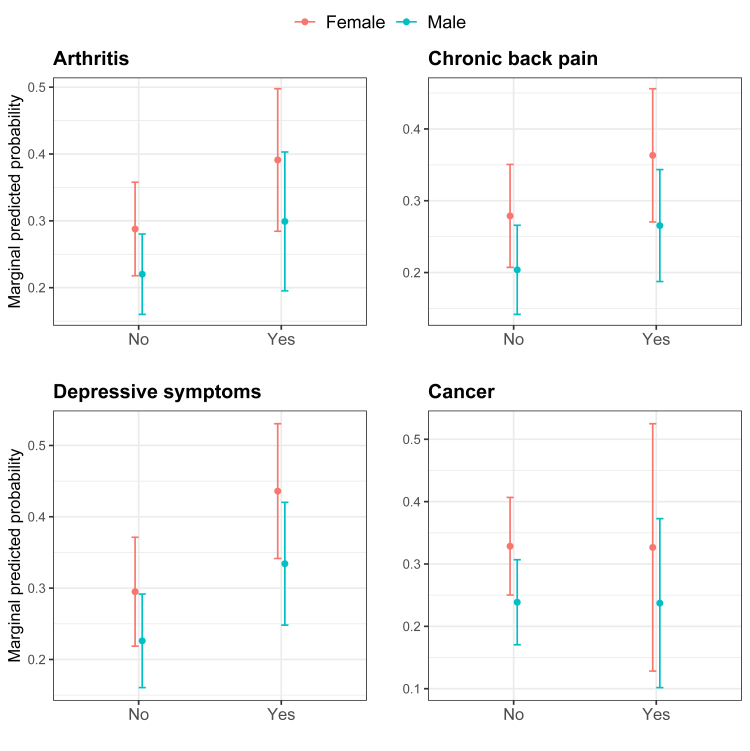
**Probability of prescription opioid use according to the presence of chronic conditions and sex, ELSI-Brazil, 2019–2020**. Marginal predictive probabilities were calculated from adjusted models (Model 1 in Table 2). Each plot shows the marginal probability of prescription opioid use (ranging from 0 to 1) by presence of the chronic conditions (yes/no) and sex.

Marginal predicted probabilities of prescription opioid use from Table 2 (*model 1*) for each chronic condition and by sex are depicted in Fig. 1. The predicted probability of prescription opioid use was consistently higher for female participants vs. male (Fig. 1).

**Fig. 2:**
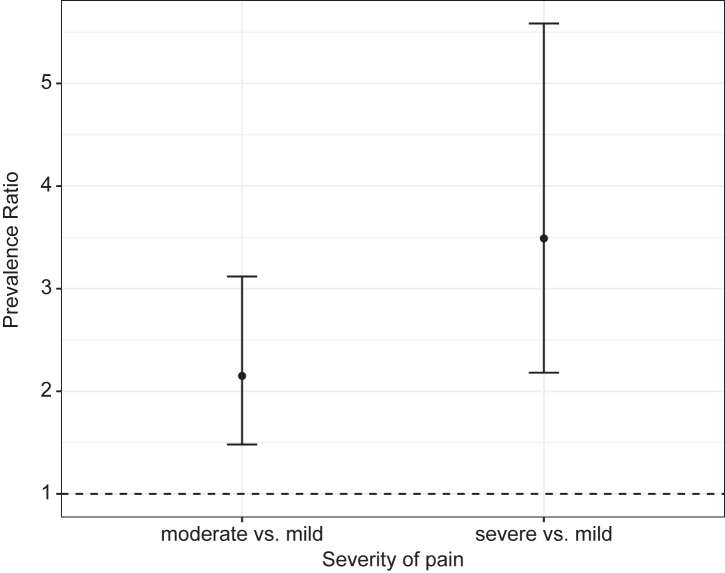
**Prevalence ratio of prescription opioid use among those who suffer from recurrent pain by severity of pain, ELSI-Brazil, 2019–2020**. Prevalence ratios were adjusted for covariates (binary sex, age, education, household income categorized into quintiles, urban/rural, and region of residence).

Finally, pain severity was associated with prescription opioid use; individuals reporting severe and moderate pain were three and two times more likely to use prescription opioids, respectively, compared to those who reported mild pain (Fig. 2).

## Discussion

In this study, based on a nationally representative sample of Brazilian older adults, we found high self-reported use of prescription opioids among those who suffer from recurrent pain. We also found that experience of pain was higher among female participants (vs. male), individuals at the bottom (vs. top) of the income distribution, and individuals with arthritis, chronic back pain, depressive symptoms, and those with a history of falls or hospitalizations compared with their counterparts. Among those who reported pain, prescription opioid use was higher among female and single (vs. married/cohabiting) individuals. The prevalence of opioid use was higher among individuals with arthritis, chronic back pain, and depressive symptoms after adjusting for covariates. Among those who suffer from recurrent pain, people who experience severe and moderate pain were three and two times more likely to use prescription opioids, respectively, compared to those who experience mild pain.

Regarding the prevalence of pain in the population, our findings are broadly consistent with previous studies in different populations. A study using survey data from 17 countries, found that the prevalence of chronic pain was 45% and 31% among women and men, respectively.^30^ A systematic literature review found a mean prevalence of pain of about 30% in low- and middle-income countries.^31^ However, systematic reviews report a wide range of prevalence rates in different population groups.^32^ Comparisons across countries and surveys are challenging due to different definitions of pain used, different ways to collect information and variations in surveys, and a preponderance of studies focused on pain caused by specific conditions such as lower back pain.^31^^,^^32^ In our study pain was defined as recurrent pain rather than chronic pain, i.e., pain that lasts for three months or more, because our data did not include the length of time related to the experience of pain. Thus, our results are not directly comparable to studies measuring chronic pain. Nonetheless, our study highlights the significant burden of pain in the older adult population in Brazil. Globally, lower back pain, headache disorders, and depressive disorders are the top three leading causes of years lived with disability (YLDs); together, these conditions resulted in an estimated 162 million YLDs globally in 2017.^33^ Our study also highlights the association between some of these conditions and experience of pain in the older adult population in Brazil.

We also identified important variations in the prevalence of pain across different population groups, which was consistent with the literature. For example, pain was more prevalent among low socioeconomic status (SES) groups,^6^^,^^34^^,^^35^ among women,^6^^,^^30^^,^^36^ and among those who have chronic conditions.^7^^,^^37^ However, our study did not find variation in prevalence of pain by age. Our data included only the population 50 and older; thus, the prevalence shown is likely higher than that in the general population, but among those 50 and older, age was not associated with pain. Higher prevalence of pain among low-income groups is likely related to higher prevalence of other conditions and higher occupational exposures among low SES vs. high SES groups.^9^^,^^32^ However, in our study, low-income groups did not have a higher prevalence of prescription opioid use compared to high-income groups. Instead, high-income groups reported similar use of opioid despite having lower prevalence of pain, compared to low-income groups. This pattern could be due to overutilization of prescription opioids among high-income individuals or unmet need for treatment among low-income individuals, who may face barriers to healthcare access despite the existence of a national public healthcare system in Brazil.

Our study shows a high prevalence of prescription opioid use; 30% of people who reported recurrent pain said they used prescription opioids in the past three months. This prevalence is much higher than rates reported by other studies using data from nationally representative surveys in Brazil.^22^^,^^25^ For example, in 2014 a national survey of medication use found a 0.8% prevalence of prescription opioid among people 60 and older. One reason for this difference is related to the composition of the population, since we calculated opioid use among those who reported recurrent pain. In this scenario, if we assume that all participants who did not report recurrent pain in ELSI-Brazil were non-users of prescription opioids (which likely underestimates the true use in the target population), the prevalence of opioid use is still high (10%). If at least a small portion of the participants who did not report recurrent pain used prescription opioids, the true prevalence of use in the target population is higher than 10%.

These findings are concerning because prescription opioid use seems to be much higher in this population compared to previous data from Brazil. Instead numbers are somewhat comparable to that of populations in the US.^36^^,^^38^^,^^39^ For example, in a US-based study, a third of people with chronic pain reported use of prescription medication for pain.^36^ However, it is important to keep in mind that comparisons across studies are challenging due to the potential for underreporting, variation in the ascertainment of the outcome, and groups of populations included, e.g. overall population^38^^,^^39^ vs. those who reported chronic pain.^36^ In addition, ELSI-Brazil did not collect data on frequency and duration of use, which are important aspects directly related to the risk of developing substance use disorders. It is possible that many respondents in our study used prescription opioids for a short period of time,^25^^,^^26^ which is less concerning though no less relevant to track.

Despite these challenges in measurement, our findings point to some concerning patterns. For example, we found that 12% and 26% of people reporting mild and moderate pain also reported prescription opioid use. In addition, we found that people with chronic conditions such as arthritis and depressive symptoms were 36% and 48% more likely to use prescription opioids than their counterparts without a chronic condition. Data on the specific reason(s) for which opioids were prescribed were not available, thus we cannot make a direct connection between the conditions/events reported and the use of opioids. It is possible that the medication was prescribed for an acute and limited event of pain. In fact, previous studies show that a large share of prescription opioids in Brazil are used for acute pain episodes.^25^ More data is needed to assess the frequency and duration of use and the specific situation in which the drug was prescribed.

We found that combinations of chronic diseases change the magnitude of the association between particular exposures and opioid use. This pattern indicates a complex relationships between these conditions. For example, the fact that the association between arthritis and opioid use reduces in magnitude in the model that includes depressive symptoms could mean that depression is a mediator in the pathway between arthritis and opioid use. Or in the case of chronic back pain, once the model is adjusted for arthritis, the association moves closer to the null, indicating that arthritis is likely to be driving opioid use in patients who also report chronic back pain. Depressive symptoms had the largest magnitude of association with opioid use. This pattern is concerning but in no way surprising. Depression is a common comorbidity among people with chronic pain.^37^ Presence of depressive symptoms may also indicate a more severe or advanced case of the chronic condition(s).

There is growing concern about the increased use of prescription opioids in Brazil and other low- and middle-income countries.^40^ Opioid sales have declined in the US and Canada due to updated guidelines to treat pain and a broader set of policies to better regulate the use of these drugs.^41^, ^42^, ^43^ This could lead to an increased focus of pharmaceutical companies on low- and middle-income countries. In fact, companies have published guidance documents to promote the use of prescription opioids for the treatment of chronic pain with a focus on Latin America, South Africa, and India.^40^ Pain management guidelines are critical to addressing the burden of pain in the population, but these guidelines should be developed independently from private interests and based on solid evidence of effectiveness and safety.^44^ Increased availability of highly addictive prescription opioids without adequate surveillance or training of providers would lead to increased opioid harm in these countries.

### Limitations

A limitation of this study is the use of self-reported measures of opioid use. On the one hand, people might underreport the use of analgesic opioids if they associate these drugs with addiction or stigma. On the other hand, since the interviewers did not check medication bottles, respondents might have mistakenly reported the use of other prescription pain medication that are not opioid based, even though the question included a list of opioid medications. In addition, respondents were not asked which specific medication was used, thus we cannot differentiate between weak (e.g., codeine) and strong (e.g., oxycodone) opioids. Another limitation was the skip pattern of the survey, i.e., only participants who reported recurrent pain answered the opioid use question. This issue prevents us from calculating prescription opioid use among those who did not report frequent pain, but future waves of the study will address this issue extending the opioid question to all participants. We also lack information about the duration and frequency of use and the reason(s) for using prescription opioids. This information is critical to assess the risk of developing opioid use disorders and should be investigated in future studies.

In conclusion, recurrent pain was reported by a sizable portion of the older adult population in Brazil, and among those, prescription opioid use was high. Considering the growing prevalence of chronic conditions associated with pain in Brazil and the growing use of prescription opioids, opioid misuse has the potential to become a public health problem in the future. Brazil, like other Latin American countries, needs robust surveillance systems to monitor opioid prescribing behavior and opioid dispensing, with a focus on groups that might be at an increased risk of developing opioid use disorders such as individuals who suffer from chronic pain. Such systems would help identify overprescribing of opioids and support initiatives to prevent opioid misuse in the population.

## Contributors

All authors (PHM, MFLC, and AILF) conceptualized the study. PHM performed data analysis and wrote the first draft. All authors discussed the results and contributed to the final manuscript. All authors had direct access to the data. All authors were responsible for the decision to submit the manuscript.

## Data sharing statement

Data and documentation are freely available to researchers upon registration with the ELSI-Brazil project at https://elsi.cpqrr.fiocruz.br/en/register/.

## Declaration of interests

The authors declare no conflict of interests.

## Appendix A Supplementary data

Supplementary Material Table S1

Abstract in Portuguese
