# Supplementary material for: Prevalence of pain and use of prescription opioids among older adults: results from the Brazilian Longitudinal Study of Aging (ELSI-Brazil)
Source: Lancet Reg Health Am. 2023 Mar 1;20:100459. doi: 10.1016/j.lana.2023.100459 (PMC9996352; doi:10.1016/j.lana.2023.100459)
Supplement: Supplementary Material Table S1 [file mmc1.docx]

**Table S1 – Proportion of ELSI-Brazil participants with complete data in the second wave, by demographic and socioeconomic characteristics, and presence of chronic conditions and events related to pain.**

|  | **Proportion of participants included in sample (complete data)** | **Proportion participants with at least on variable missing** |
| --- | --- | --- |
| **Sex** |  |  |
| Female | 93.1 | 6.9 |
| Male | 93.0 | 7.0 |
| **Age group** |  |  |
| 50-59 | 92.8 | 7.2 |
| 60-69 | 94.1 | 5.9 |
| 70-79 | 92.3 | 7.7 |
| 80+ | 92.1 | 7.9 |
| **Education** |  |  |
| 0-4 years | 94.5 | 5.5 |
| 5-8 years | 92.4 | 7.6 |
| 9-11 years | 91.6 | 8.4 |
| 12 or more | 88.4 | 11.6 |
| **Marital status** |  |  |
| Single | 91.5 | 8.5 |
| Married/cohabiting | 94.0 | 6.0 |
| Divorced | 91.0 | 9.0 |
| Widowed | 92.0 | 8.0 |
| **Household income** |  |  |
| Q1 (lowest quintile) | 97.6 | 2.4 |
| Q2 | 98.6 | 1.4 |
| Q3 | 98.7 | 1.3 |
| Q4 | 99.1 | 0.9 |
| Q5 (highest quintile) | 99.1 | 0.9 |
| **Region** |  |  |
| North | 95.5 | 4.5 |
| Northeast | 95.7 | 4.3 |
| Southeast | 89.3 | 10.7 |
| South | 94.8 | 5.2 |
| Center-West | 98.7 | 1.3 |
| **Residence** |  |  |
| Urban | 92.1 | 7.9 |
| Rural | 98.3 | 1.7 |
| **Arthritis** |  |  |
| Yes | 95.1 | 4.9 |
| No | 93.6 | 6.4 |
| **Chronic back pain** |  |  |
| Yes | 95.9 | 4.1 |
| No | 92.5 | 7.5 |
| **Depressive symptoms** |  |  |
| Yes | 94.7 | 5.3 |
| No | 93.4 | 6.6 |
| **Cancer** |  |  |
| Yes | 91.4 | 8.6 |
| No | 93.6 | 6.4 |
| **Fall (last 12 months)** |  |  |
| Yes | 95.2 | 4.8 |
| No | 93.4 | 6.6 |
| **Hospitalizations** |  |  |
| None | 93.1 | 6.9 |
| One | 92.6 | 7.4 |
| Two or more | 92.8 | 7.2 |
