# Supplementary material for: Prevalence of pain and use of prescription opioids among older adults: results from the Brazilian Longitudinal Study of Aging (ELSI-Brazil)
Source: Lancet Reg Health Am. 2023 Mar 1;20:100459. doi: 10.1016/j.lana.2023.100459 (PMC9996352; doi:10.1016/j.lana.2023.100459)
Supplement: Abstract in Portuguese [file mmc2.docx]

**Titulo:** Prevalência de dor e uso de analgésicos opioides em idosos: resultados do Estudo Longitudinal de Saúde dos Idosos Brasileiros (ELSI-Brasil).

**Introdução:** A dor tem um impacto considerável na qualidade de vida das pessoas. O uso de analgésicos opioides no tratamento da dor esta associado ao risco de dependência desses medicamentos e mortes por overdose. O objetivo desse estudo é caracterizar a prevalência de dor e o uso de analgésicos opioides entre idosos brasileiros.

**Materiais e métodos:** Utilizamos dados da segunda onda do Estudo Longitudinal de Saúde dos Idosos Brasileiros (ELSI-Brasil), 2019-2020 (média de idade=63,3; 54,4% sexo feminino). As variáveis respostas foram: (1) experiencia de dor recorrente, e (2) uso de analgésicos opioides nos últimos 3 meses entre aqueles que relataram dor. As variáveis de exposição incluíram condições de saúde, histórico de quedas e de hospitalização.

**Resultados:** A prevalência de dor (n=9.234) foi de 36,9% (IC 95%: 32,6-41,4). A dor foi relatada com maior frequência por participantes do sexo feminino, pessoas de baixa renda, pessoas com diagnostico prévio de artrite, problema crônico de coluna, sintomas depressivos, histórico de quedas e hospitalizações. A prevalência do uso de opioides entre os que relataram dor (n=3.350) foi de 30% (IC 95%: 23,1-38,0). A prevalência do uso de opioides foi maior entre indivíduos do sexo feminino e pessoas solteiras. Em modelos ajustados, artrite, problema crônico de coluna e sintomas depressivos foram associados ao uso de analgésicos opioides.

**Interpretação:** O uso de analgésicos opioides foi relatado por uma parcela considerável dos idosos que sofrem com dor no Brasil. Em um contexto de consumo crescente de analgésicos opioides, a dependência desses medicamentos pode aumentar no futuro. A vigilância de analgésicos opioides é fundamental no monitoramento desses medicamentes com o objetivo de evitar o uso inadequado dessas substancias e suas consequências devastadoras na saúde da população.

**Financiamento:** O ELSI-Brasil foi financiado pelo Ministério da Saúde do Brasil.
